# Supplementary material for: WordCommentsAnalyzer: A windows software tool for qualitative research
Source: F1000Res. 2018 Sep 4;7:536. Originally published 2018 May 3. [Version 2] doi: 10.12688/f1000research.14819.2 (PMC6137411; doi:10.12688/f1000research.14819.2)
Supplement: Supplementary file 3 [file f1000research-7-17614-s0002.tgz › b2a79496-4ada-47c4-9682-a99ce867836d.zip!/Eight Iranian Health Journals/The Journal of Toloo-e-Behdasht_22.docx]

© The Journal of Toloo-e-Behdasht. This work is licensed under CC BY 4.0 (https://creativecommons.org/licenses/by/4.0/).

Volume & Issue: دوره 13 - شماره 5

Title: ارزیابی ارتعاش تمام بدن در رانندگان خودرو های سنگین معدن مجتمع گل گهر سیرجان

http://tbj.ssu.ac.ir/article-1-1283-fa.html

مقدمه: کلیه خودرو ها انسان را در معرض ارتعاش تمام بدن با شدت های مختلفی قرار می دهند. ارتعاش تمام بدن می تواند باعث سلب آسایش و ایجاد اختلالات اسکلتی- عضلانی در افرادی که با آن مواجه هستند، شود. خودرو های سنگین معدن، با توجه به محیط کار و ظرفیت کاری آنها و اینکه خودرو های قدرتی هستند ارتعاش زیادی تولید می کنند و به تبع آن اپراتور های آنها نیز در مواجهه با این عامل زیان آور فیزیکی قرار می گیرند. هدف این مطالعه ارزیابی ارتعاش تمام بدن در رانندگان خودرو های سنگین معدن مجتمع گل گهر سیرجان می باشد. روش بررسی: این مطالعه از نوع مطالعات مقطعی و در خودروهای سنگین بخش معدن، مجتمع گل گهر سیرجان انجام گردید. در این مطالعه ارتعاش تمام بدن در 92 دستگاه از خودرو های معدن مطابق با الزامات استاندارد (1997)- 1- 2631ISO مورد بررسی قرار گرفتند. برای سنجش ارتعاش تمام بدن از دستگاه ارتعاش سنج مدل 958SVAN استفاده شد. اطلاعات به دست آمده با نرم افزار 16SPSS مورد تجزیه و تحلیل قرار گرفت. یافته ها: میانگین شتاب معادل کلی تمام بدن خودرو ها نشان داد که در خودرو های گریدر با 179/2 متر بر مجذور ثانیه و پس از آن بلدوزر ها با 738/1 متر بر مجذور ثانیه بیشترین مقدار ارتعاش را دارند. خودرو های دریل با 479/0 متر بر مجذور ثانیه کمترین مقدار شتاب معادل کلی تمام بدن را در بین همه مدل ها دارا می باشند. در مقایسه شتاب معادل کلی خودرو ها با استاندارد مربوطه، مشخص گردید که بجز دریل ها مقدار شتاب معادل کلی در همه خودرو ها بیش از استاندارد حد مراقبت 8 ساعته می باشد. نتیجه گیری: این مطالعه نشان داد ارتعاش در اکثر خودرو های سنگین معدن بسیار بیشتر از حد استاندارد و حتی از حد مراقبت نیز بالاتر بود. لذا با توجه به اثرات نامطلوب بهداشتی و فیزیولوژیکی در مواجهه با ارتعاش، اقدامات اصلاحی در خصوص سیستم تعلیق خودرو ها و وضعیت صندلی آنها و همچنین کنترل های مدیریتی برای رانندگان این خودرو ها ضروری می باشد. این مقاله برگرفته از پایان نامه کارشناسی ارشد رشته مهندسی بهداشت حرفه ای دانشگاه علوم پزشکی کرمان می باشد.

Title: آلودگی میکروبی و قارچی هوای کلینیکها و مطب‌های دندانپزشکی شهر کرمان

http://tbj.ssu.ac.ir/article-1-1607-fa.html

مقدمه: وجود آلودگی باکتریایی در اتمسفر محیط کلینیکها و مطبهای دندانپزشکی مایه نگرانی است.در این مورد بیشترین عامل آلودگی ذرات معلقی است که عموما" حاوی میکروارگانیسمهای مختلف بیماریزا بوده ودر هوای داخل کلینیکها پراکنده است.به منظور ارائه راهکارهایی جهت ارتقاء کیفیت هوای کلینیکها و مطبهای دندانپزشکی کرمان در این تحقیق به بررسی وضعیت هوای این مراکز از نظر آلودگی میکروبی و قارچی پرداخته شد. روش بررسی: مطالعه توصیفی - مقطعی است که در بازه زمانی 1391لغایت 1392در کلینیکها و مطبهای دندانپزشکی شهر کرمان انجام شد. از 14 کلینیک دندانپزشکی موجود در شهر، دو کلینیک دندانپزشکی دولتی، سه کلینیک خصوصی، یک کلینیک نیمه خصوصی، یک کلینیک خیریه و از 143 مطب دندانپزشکی موجود،52 مطب انتخاب شد. نمونه گیری با روش تصادفی و نمونه برداری با روش ته نشینی انجام گرفت. محیط کشت مورد استفاده، نوترینت آگار، بلادآگار و نوترینت آگار به همراه کلرامفنیکل و محیط کشت سابرد دکستروز جهت قارچها بود. از رنگ آمیزی گرم و محیط کشتهای بیو شیمیایی و اسلاید کالچر جهت تشخیص استفاده شد. مشخصات محل نمونه گیری از قبیل: مساحت، تعداد یونیتها، حجم فضا، زمان استفاده، تردد، نوع خدمت و استفاده از توربین نیز یادداشت شد. از آزمون آماره کای دو وآزمون دقیق فیشر جهت قضاوت آماری استفاده شد. یافته ها: در این مطالعه 89 درصد از نمونه های مورد بررسی آلودگی میکروبی و قارچی داشتند. گونه های یافت شده میکروبی شامل کوکسی های گرم مثبت (2/60 درصد)، کوکسی های گرم منفی( 1/24 درصد)، باسیل گرم مثبت( 2/11 درصد)، باسیل گرم منفی 4/2 درصد و همچنین قارچها(2/1 درصد) بودند. بین میزان آلودگی، تعداد مراجعین، فضا(کلینیک ها و مطب ها)، نوع کاربری بخش ها وشیفت کاری و غیرکاری ارتباط معنی دار آماری دیده شد( 001/0 > p). نتیجه گیری: درجه آلودگی میکروبی هوای کلینیکها و مطبهای دندانپزشکی کرمان بر اساس رتبه بندی AMI (Air Microbial Index) در وضعیت بد قرار داشت. بهبود سیستم تهویه کلینیکها و مطبهای دندانپزشکی برای ارتقاء رتبه بندی از بد به خوب ضرورت دارد.

Title: بررسی نگرش، عقاید رفتاری و ارزشیابی پیامد رفتاری پدران شهر یزد در مورد پیشگیری از گرایش فرزندان به مواد اعتیاد آور

http://tbj.ssu.ac.ir/article-1-1285-fa.html

مقدمه: سوءمصرف مواد و اعتیاد در ایران در حال گسترش است. با کاهش سن شروع اعتیاد به نظر می‌رسد پیشگیری از مواجهه کارآمدترین روش باشد. از آنجا که خانواده مهم‌ترین نهاد اجتماعی در جامعه پذیری و تربیت فرزندان است، این مطالعه با هدف تعیین نگرش، عقاید رفتاری و ارزشیابی پیامد رفتاری پدران شهر یزد در مورد پیشگیری از گرایش فرزندان به مواد اعتیاد آور انجام گرفت. روش بررسی: این مطالعه توصیفی- تحلیلی و به روش مقطعی بود. جامعه پژوهش پدران شهر یزد بودند که در یک نمونه 180 نفری به روش نمونه گیری تصادفی چند مرحله‌ای انتخاب شدند و مورد مطالعه قرار گرفتند. ابزار جمع آوری اطلاعات، پرسشنامه محقق ساخته دارای روایی و پایایی معتبر در سه بخش؛ جمعیت شناختی و زمینه‌ای با هفت سوال ، چهار گویه نگرش (81/0=α) و محدوده امتیاز 28-4، پانزده گویه عقاید رفتاری (89/0=α) و محدوده امتیاز 105-15 و پانزده گویه ارزشیابی پیامد رفتار (82/0=α) و محدوده امتیاز 105-15 بود که به صورت خود گزارش دهی تکمیل می‌شد. داده‌های جمع آوری شده توسط نرم افزار آماری SPSS-18 و با بهره گیری از آمار توصیفی و آزمون‌های تحلیلی نظیرتی تست مستقل، آنووا، آنالیز رگرسیون خطی و ضریب همبستگی پیرسون در سطح معناداری 05/0 مورد آزمون قرار گرفتند. یافته‌ها: محدوده سن شرکت کنندگان 30 الی 59 سال با میانگین 34/5±38/40 بود. میانگین نمره کسب شده از نگرش 14/5±45/19، اعتقاد رفتار 94/14±76/78 و ارزشیابی پیامد رفتار 10/9±81/81 بود. نگرش (001/0=p) و اعتقادات رفتار (050/0=p) پدرانی که صاحب خانه بودند، نگرش (001/0=p) و اعتقادات رفتار (012/0=p) پدران دارای سطح لیسانس و بالاتر از امتیاز بیشتری برخوردار بود. میان نگرش و اعتقادات رفتاری (001/0>p و368/0=r)، نگرش و ارزشیابی پیامد رفتاری (001/0>p و304/0=r) و عقاید رفتاری و ارزشیابی پیامد رفتاری (001/0>p و792/0=r) همبستگی وجود داشت. همچنین آزمون رگرسیون نشان داد عقاید رفتاری و ارزشیابی پیامد رفتاری هرکدام به ترتیب 44 و 31 درصد از واریانس نگرش را پیشگویی می نمودند. نتیجه گیری: نگرش، عقاید رفتاری و ارزشیابی پیامد رفتاری پدران در خصوص انجام رفتارهای پیشگیری کننده از گرایش مصرف مواد اعتیاد آور در فرزندان مطلوب بود از اینرو اهمیت و ضرورت اجرای برنامه پیشگیرانه از طریق پدران در این خصوص مورد تاکید قرار می‌گیرد. این مقاله برگرفته از پایان نامه کارشناسی ارشد رشته دانشگاه علوم پزشکی شهید صدوقی یزد می باشد.

Title: آنالیز مکانی کانون های لیشمانیوز جلدی روستایی در استان قم با استفاده از سیستم اطلاعات جغرافیایی(GIS)

http://tbj.ssu.ac.ir/article-1-1286-fa.html

مقدمه: لیشمانیوز جلدی روستایی یکی از معضلات مهم بهداشتی در بسیاری از مناطق ایران از جمله استان قم می باشد. این تحقیق با هدف بررسی روند بروز لیشمانیوز جلدی، شناسایی کانون های پرخطر بیماری در استان قم با استفاده از سیستم اطلاعات جغرافیایی صورت گرفت. روش بررسی: اطلاعات بیماران مربوط به سال های 90-1373 از فرم های اطلاعات اپیدمیولوژیک لیشمانیوز جلدی مرکز بهداشت استان قم استخراج گردید .سپس بر روی نقشه دیجیتال شهری و روستایی استان با مقیاس 50000/1 مکان یابی شدند. تعمیم یافته ها به نقشه در سطح روستا انجام شد و جمعیت هر روستا بر اساس جمعیت برآورد شده در خانه بهداشت روستا تعیین شد. کانون ها بر اساس میزان بروز محاسبه شده، شناسایی شدند. برای تجزیه و تحلیل یافته ها از آزمون کای اسکور و ترسیم نقشه ها از نرم افزار Arc GIS 3/9 استفاده گردید. یافته ها : در مجموع ،اطلاعات مربوط به 1663 بیمار ثبت گردید. کانون های اصلی بیماری در نواحی شرق و شمال شرق استان و شامل روستا های واقع در بخش های مرکزی و کهک بودند. تعداد 1555 (5/93%) مورد از بیماران مربوط به روستاهای بخش مرکزی بودند. روستاهای صیدآباد و جعفرآباد به ترتیب با درصد شیوع 78/67 و 67/66 ، کانون های فعال تری بودند. کانون های جدید در روستا های دولت آباد، اسلام آباد و مومن آباد در حال شکل گیری بودند. کانون هایی نظیر روستاهای حسین آباد و صرم آمار موارد آنها رو به کاهش بود (01/0 p<). نتیجه گیری: شناسایی تجمع مکانی لیشمانیوز و اولویت بندی عوامل موثر در چرخه انتقال بیماری با استفاده از نقشه های خروجی از سیستم اطلاعات جغرافیایی (GIS) قابل دستیابی بوده و این نرم افزار می تواند به عنوان ابزاری کارآمد در جهت برنامه ریزی و تصمیم گیری برای انجام اقدامات پیشگیرانه در مناطق پرخطر بیماری به کار گرفته شود.

Title: بررسی وضعیت ویتامین D در مبتلایان به پرفشاری خون در مرکز بهداشتی درمانی آزادشهر یزد سال 1392

http://tbj.ssu.ac.ir/article-1-1287-fa.html

مقدمه:کمبود ویتامین D یکی از مهمترین عواملی است که در پاتوژنز و پیشرفت پرفشاری خون مؤثر دانسته شده است. هدف از این مطالعه بررسی وضعیت ویتامین D در افراد مبتلا به پرفشاری خون در شهر یزد بوده است. روش بررسی: افراد شرکت کننده در این مطالعه توصیفی– مقطعی 56 نفر شامل 35 زن و21 مرد 50-25 ساله دارای پرونده فشار خون در مرکز بهداشتی درمانی شهری آزادشهر یزد بودند که جهت تشخیص قطعی پرفشاری خون (دارا بودن فشار خون سیستولی بیشتر یا مساوی 140 میلی متر جیوه و فشار خون دیاستولی بیشتر یا مساوی 90 میلی متر جیوه) فشار خون آنها مجددا اندازه گیری گردید. جمع آوری داده ها به وسیله نمونه های خونی جهت بررسی غلظت های سرمی 25- هیدروکسی ویتامین D ، پاراتورمون، کلسیم، سدیم، پتاسیم، فسفر و منیزیم انجام گرفت.کمبود ویتامین Dبه صورت سطح سرمی 25 – هیدروکسی ویتامین D کمتر از 30 نانوگرم در میلی لیتر تعریف شد. در این مطالعه ،کمبود خفیف ویتامین D ، به صورت سطح سرمی 25 – هیدروکسی ویتامین D بین 20 تا 9/29 نانوگرم در میلی لیتر ، کمبود متوسط بین 10 تا 9/19 نانوگرم در میلی لیتر و کمبود شدید کمتر از 10 نانوگرم در میلی لیتر تعریف شد. یافته ها: بر اساس یافته های این مطالعه مشخص شد که 19 نفر از افرادمورد بررسی (9/33%) مبتلا به کمبود خفیف، 16 نفر مبتلا به کمبود متوسط(6/28%) و 7 نفر از آنها(5/12%) مبتلا به کمبود شدید ویتامین D بودند. تنها 14 نفر (25%) از افراد مورد بررسی وضعیت ویتامین D طبیعی داشتند.لازم به ذکر است. میانگین غلظت سرمی پاراتورمون، کلسیم، سدیم، پتاسیم، فسفر و منیزیم در تمام افراد در محدوده طبیعی قرار داشت. بین متغیر جنس یا سن و وضعیت ویتامین D افراد ارتباط معنا داری یافت نشد (05/0 P<). نتیجه گیری: بر اساس یافته های این مطالعه، فراوانی کمبود ویتامین D در افراد مبتلا به پرفشاری خون مورد بررسی بسیار قابل ملاحظه بود. علاوه بر این رفع کمبود این ویتامین و اثر آن بر پرفشاری خون قابل توصیه است. این مقاله بر گرفته از پایان نامه کارشناسی ارشد دانشگاه علوم پزشکی و خدمات بهداشتی، درمانی شهید صدوقی یزد می باشد.

Title: بررسی میزان شیوع افسردگی، اضطراب و استرس در دانش آموزان دختر دبیرستانی شهر مشهد و ارتباط آن با برخی از رفتارهای مرتبط با سلامت

http://tbj.ssu.ac.ir/article-1-1288-fa.html

چکیده مقدمه: دوره نوجوانی به دلیل تغییرات روانی، اجتماعی و زیستی یکی از بحرانی ترین دوران زندگی هرفرد به شمار می رود که می تواند عملکرد تحصیلی و شغلی را تحت تاثیر قرار دهد. از آنجایی که توجه به بهداشت روانی دانش آموزان از محورهای مهم بهداشت روانی جامعه به شمار می رود، این مطالعه با هدف تعیین ارتباط اضطراب، افسردگی و استرس با رفتارهای بهداشتی در دختران دانش آموز شهر مشهد انجام شد. روش بررسی: این مطالعه از نوع توصیفی -مقطعی می باشد که بر روی 407 نفر از دانش آموزان دختر دبیرستانی شهر مشهد و به طریق نمونه گیری احتمالی دو مرحله ای انجام شد. ‌واحدهای پژوهش پرسشنامه های مربوط به مشخصات فردی، خانوادگی، رفتارهای بهداشتی و مقیاس استاندارد دس 21 را تکمیل نمودند. داده ها با استفاده ازنرم افزارآماری SPSSنسخه 14و آزمونهای آماری ‌انجام شد. یافته ها: یافته ها نشان داد که 5/56% از دانش آموزان 16-14سال، 3/66% شاخص توده بدنی 99/24-5/18 کیلوگرم بر متر مربع، 6/36 درصد فعالیت فیزیکی و 2/83، با دود سیگار تماس داشتند. 7/21% افسردگی، 3/24%اضطراب و 8/21% استرس شدید و بالاتر داشتند که سن با اضطراب و استرس همبستگی مثبت و با افسردگی همبستگی منفی داشت. درحالیکه بین میزان اضطراب، افسردگی و استرس با سایر متغیرها همبستگی دیده نشد. نتیجه گیری: باتوجه به بالابودن میزان افسردگی، اضطراب و استرس در دانش آموزان دختر دبیرستانهای شهر مشهد و نقش مهم دختران در جامعه بنابراین، انجام برنامه های مختلف مشاوره ای از جمله مشاوره های روانی و تحصیلی به منظور ارتقاء سلامت روانی دانش آموزان و ارجاع دانش آموزان دارای علائم شدیدتر به متخصصین و روان پزشکان جهت پیگیری درمان اختلالات لازم و ضروری است.

Title: بررسی وضعیت رفتارهای محافظت کننده در برابر نور خورشید جهت پیشگیری از سرطان پوست و موانع انجام آن در کشاورزان شهرستان کازرون در سال 1392

http://tbj.ssu.ac.ir/article-1-1289-fa.html

مقدمه: کشاورزان به علت تماس مدوام با نورخورشید در معرض ابتلا به سرطان پوست هستند و رفتارهای محافظت کننده در برابر نور خورشید اساسی ترین اقدام در پیشگیری از آن به شمار می رود. مطالعه حاضر به منظور تعیین وضعیت به کارگیری رفتارهای محافظت کننده در برابر نور خورشید و عوامل مرتبط با آن در کشاورزان شهرستان کازرون انجام شد. روش بررسی: در این مطالعه توصیفی مقطعی 300 نفر از کشاورزان شهرستان کازرون در استان فارس که به روش خوشه ای از بین 8 روستا در بهار سال 1392، انتخاب شده بودند، مورد مطالعه قرار گرفتند. ابزار جمع آوری اطلاعات پرسشنامه ای محقق ساخته با روایی و پایایی قابل قبول بود که شامل: اطلاعات دموگرافیک، سؤالاتی که عملکرد کشاورزان را درخصوص به کارگیری رفتارهای محافظت کننده در برابر نور خورشید را اندازه گیری می نمود و سؤالاتی درخصوص علل عدم به کارگیری این رفتارها و منبع کسب اطلاعات درخصوص سرطان پوست و راه های پیشگیری از آن بود، که به روش مصاحبه حضوری با کشاورزان تکمیل گردید و با کمک نرم افزار SPSS مورد تجزیه و تحلیل قرار گرفت. یافته ها: رفتارهای محافظت کننده در برابر نور خورشید در کشاورزان بالاتر از متوسط بود. بیشترین رفتار گزارش شده"پوشیدن لباس هایی که قسمت بیشتری از بدن را می پوشاند" و کمترین رفتار اتخاذ شده " استفاده از کرم ضدآفتاب" بود. مهم ترین علت عدم به کارگیری رفتارهای محافظت کننده، گرانی وسایل محافظت کننده عنوان گردید. ارتباط معنی داری بین سن، جنس، میزان تحصیلات، میزان درآمد، بعد خانوار و سابقه آفتاب سوختگی با رفتارهای محافظت کننده را مشاهده نشد(05/0>p). سابقه ابتلا به آفتاب سوختگی احتمال به کارگیری رفتارهای محافظت کننده را 048/2 برابر افزایش می دهد. نتیجه گیری: با توجه متوسط بودن وضعیت به کار گیری رفتارهای محافظت کننده در برابر نور خورشید در کشاورزان و با درنظر گرفتن این نکته که رفتار مبتنی بر آگاهی و گرایش می باشد، جهت ارتقای رفتارهای محافظت کننده در برابر نور خورشید و کاهش خطر ابتلا به سرطان پوست، لزوم تدوین برنامه آموزشی با توجه به یافته های این تحقیق پیشنهاد می گردد.

Title: بررسی چالش رفتار من سانی در انتصاب مدیران مورد مطالعه : مدیران دانشگاه علوم پزشکی شیراز

http://tbj.ssu.ac.ir/article-1-1290-fa.html

چکیده مقدمه: انتخاب و استخدام کارکنان و مدیران یکی از فعالیت‌های حیاتی سازمان‌های امروزی است و در تمام سطوح، استخدام‌کنندگان سعی در انتخاب بهترین گزینه‌ها دارند؛ در این راستا، اثر من سانی از چالش هایی است که به ثبات مدیریتی صدمه می‌زند. این اثر به پدیده‌ای اشاره دارد که در آن شخص انتخاب کننده، فردی را انتخاب می‌کند که دارای صفات دموگرافیک و ویژگی‌های نگرشی مشابه با خودش باشد. مطالعه حاضر با هدف شناخت عوامل تاثیرگذار بر رفتار من سانی و چالش های مرتبط با آن انجام گردید. روش بررسی: این پژوهش از نوع مطالعات مقطعی- توصیفی است که جمعیت هدف آن را کلیه مدیران ارشد و میانی دانشگاه علوم پزشکی شیراز تشکیل دادند. ابزار جمع آوری داده ها پرسشنامه ای با چهار بعد بود که علاوه بر ضریب آلفای کرونباخ بیش از 7/0 برای هر بعد، روایی محتوای آن طبق نظر متخصصین و اساتید علوم رفتاری، مورد تایید قرار گرفت. برای تجزیه و تحلیل اطلاعات، از ضریب همبستگی پیرسون و نرم افزار آماری SPSS نسخه 18 استفاده گردید. یافته ها: مطابق با یافته‌های پژوهش که با مشارکت 60 نفر از مدیران دانشگاه علوم پزشکی شیراز به دست آمد، خود شیفتگی مدیران با بروز اثر من سانی همبستگی داشته(805/0r=) و همچنین ارتباط مثبتی میان اثر من سانی با بروز گروه‌اندیشی در گروه‌های تصمیم‌گیری سازمانی مشاهده گردید(875/0r=) از سوی دیگر بین اثر من سانی با تضاد کارکردی همبستگی منفی وجود داشت(685/0-r=). نتیجه گیری: بر اساس یافته های مطالعه، پیامدهای مخرب اثر من سانی از طریق خودشیفتگی، گروه اندیشی و اجتناب از تضاد کارکردی می تواند به عملکرد مدیران دانشگاه علوم پزشکی شیراز لطمه بزند که برای نیل به اهداف سازمان، می بایست چنین رفتارهایی را مورد شناسایی قرار داده و از آن اجتناب نمود.

Title: بررسی کارایی پودر گل گیاه تلخه در حذف رنگ راکتیو قرمز 198 از محیط‌های آبی

http://tbj.ssu.ac.ir/article-1-1291-fa.html

مقدمه: فاضلاب‌های رنگی صنایع نساجی معمولاً حاوی مواد سمی و پایدار در محیط‌ زیست می‌باشند. لذا در طی سال‌های گذشته کاربرد روش‌های متعددی در حذف آنها بررسی شده است. رنگ راکتیو قرمز198 از نظر ساختار جزء رنگ‌های منو آزو است که در حال حاضر در صنایع نساجی داخل کشورکاربرد بسیار گسترده‌ای دارد. هدف مطالعه حاضر، بررسی کارایی پودر گل گیاه تلخه در حذف رنگ راکتیو قرمز 198 از محیط های آبی است. روش‌ بررسی: این مطالعه بصورت تجربی و آزمایشگاهی انجام و در آن اثر pH محلول، غلظت رنگ، دوز جاذب و زمان تماس مورد بررسی قرار گرفته است.در این مطالعه غلظت رنگ به روش اسپکتروفتومتری در طول موج 518 نانومتر تعیین گردید. میزان مطابقت داده‌ها با ایزوترم‌های لانگمیر، فروندلیچ و تمکین نیز بررسی شده است. یافته‌ها: نتایج بیانگر افزایش راندمان حذف رنگ با کاهش pH، افزایش دوز جاذب و افزایش زمان می‌باشد.افزایش غلظت رنگ از 10 به 50 میلی‌گرم در لیتر در حضور 8/0 گرم در 100 میلی لیتر جاذب، 4=pH و زمان تماس 120 دقیقه منجر به کاهش راندمان حذف از 89/67% به 16/47% گردید. به علاوه ایزوترم فروندلیچ تطابق بهتری با داده‌های جذب مطالعه حاضر داشت. نتیجه‌گیری: نتایج بدست آمده از مطالعه حاضر نشان داد که پودر گل گیاه تلخه دارای راندمان بالایی در حذف رنگ راکتیو قرمز 198 است. این جاذب آماده‌سازی آسان و هزینه کمی را دربردارد و به دلیل توانایی جذب خوب، می‌توان آن را برای حذف سایر آلاینده‌های زیست محیطی نیز استفاده نمود.

Title: مقایسه سبک های فرزندپروری و سبکهای هویتی در کودکان و نوجوانان عادی و بزهکار

http://tbj.ssu.ac.ir/article-1-1292-fa.html

چکیده مقدمه: بزهکاری به عنوان یکی از مسائل اجتماعی در جوامع مختلف بصورت حاد و به اشکال پیچیده شناخته شده است. کاهش میزان بزهکاری کودکان و نوجوانان جامعه، یکی ازسیاست های کلان مورد تاکید در سالهای اخیر بوده است،کنترل و پیشگیری بزهکاری کودکان و نوجوانان می تواند به شکل مستقیم بر بالندگی وسعادت سرمایه های با ارزش انسانی تاثیر بگذارد و بستر مساعدی را برای زندگی بهتر فراهم آورد. لذااین مطالعه با هدف مقایسه سبک های فرزندپروری ، وسبک های هویتی در کودکان و نوجوانان بزهکار و عادی صورت گرفت. روش بررسی: در این مطالعه توصیفی،تحلیلی، 300 نفر کودک و نوجوان پسر(150 نفر بزهکار و 150 نفر عادی) مورد مطالعه قرار گرفتند که از مراکز کانون اصلاح وتربیت ومدارس عادی و از طریق روش نمونه گیری تصادفی خوشه ای انتخاب شدند. آزمودنی ها پرسشنامه سبک های فرزندپروری ادراک شده،پرسشنامه سبک های هویت برزونسکی و پرسشنامه اطلاعات جمعیت شناختی را تکمیل کردند . داده های جمع اوری شده با استفاده از نرم افزار SPSS و آزمون آماری آنوا،من ویتنی،ویل کاکسون مورد تجزیه و تحلیل قرار گرفتند. یافته ها: نتایج نشان داد سبک های هویت، وضعیت اقتصادی، سطح تحصیلات والدین، وضعیت اشتغال والدین، موفقیت در تحصیل و سوابق زندانی اعضاء خانواده، در دو گروه متفاوت می باشد.تفاوت معناداری بین سبک های فرزندپروری والدین دردو گروه مشاهده نشد. همچنین بین کلیه سبک های هویت نوجوانان عادی و بزهکار تفاوت معناداری وجود دارد. نتیجه گیری: ویژگی های جمعیت شناختی، وضعیت اقتصادی، نوع اشتغال والدین، سطح تحصیلات والدین، موفقیت در تحصیل، سوابق زندانی اعضاء خانواده و سبک های هویتی از عوامل اساسی موجود در بزهکاری کودکان و نوجوانان می باشد که می تواند نتایج ارزنده وراههای مؤثری را برای کمک و حمایت از این گروه از افراد جامعه باشد.

Title: ارزیابی وضعیت ارائه آموزش بهداشت دهان در مطب های خصوصی شهر یزد

http://tbj.ssu.ac.ir/article-1-1294-fa.html

مقدمه: آموزش بهداشت دهان و دندان امری ضروری در درمان و پیشگیری از پوسیدگی های دندانی و بیماری پریودنتال در نظر گرفته می شود که به روشهای متفاوت و توسط افراد مختلفی ارائه می گردد. با توجه به اینکه دندانپزشکان عمده ترین گروهی هستند که وظیفه آموزش بهداشت به بیماران را بر عهده دارند این مطالعه با هدف بررسی نحوه نگرش دندانپزشکان شهر یزد نسبت به آموزش بهداشت دهان در مطب های شخصی صورت گرفته است. روش بررسی: این مطالعه توصیفی- مقطعی با استفاده از پرسشنامه ای حاوی اطلاعات دموگرافیک و سوالاتی پیرامون روشهای مختلف آموزش بهداشت تهیه و در اختیار 151 دندانپزشک که در شهر یزد مشغول به فعالیت در مطب های شخصی می باشند قرار داده شد .داده های به دست آمده با استفاده از نرم افزار آماری16 SPSS و آزمون فیشر تجزیه و تحلیل شد. یافته ها: داده ها بیانگر آن هستند که 3/95% از کل دندانپزشکان حداقل یک مورد از وسایل بهداشت دهان را به نحوی به بیماران خود آموزش می دهند که این آموزش بیشتر شامل روش صحیح استفاده از نخ دندان و سپس مسواک زدن می باشد. فقط 8/50% دندانپزشکان به تمام بیماران آموزش می دادند. همچنین از میان روشهای پرسش شده برای آموزش بهداشت شامل آموزش شفاهی،آموزش با استفاده از ماکت، نصب پوستر، بروشورهای آموزشی، معرفی کتاب، آموزش توسط بهداشتکار دهان در مطب، پخش برنامه های صوتی- تصویری؛ استفاده از ماکت دهان متداول تر از سایر روشها بوده است. نتیجه گیری:یافته های حاصل از این مطالعه نشان می دهند که اکثریت دندانپزشکان شهر یزد آموزش بهداشت به بیماران را به نحوی در برنامه خود قرار داده اند. بادر نظر داشتن نقش کلیدی دندانپزشکان درآموزش بهداشت دهان و دندان برای ارتقا بهداشت و سلامت دهان افراد جامعه نیاز است که این برنامه گسترده تر و یکپارچه تر شود.

Title: بررسی رابطه سواد سلامت و خودکارآمدی با سلامت دهان و دندان خودگزارش شده در دانشجویان

http://tbj.ssu.ac.ir/article-1-1293-fa.html

چکیده مقدمه: سلامت دهان و دندان جزئی از سلامت عمومی بوده و یک عامل تعیین کننده برای بهبود کیفیت زندگی محسوب می شود. هدف از پژوهش حاضر تعیین رابطه سواد سلامت دهان و خودکارآمدی بهداشت دهان با سلامت دهان و دندان خودگزارش شده در دانشجویان بود. روش بررسی: پژوهش حاضر یک مطالعه مقطعی از نوع همبستگی بود. 281 نفر از دانشجویان کارشناسی دانشگاه اردکان، براساس فرمول محاسبه حجم نمونه کوکران، به صورت نمونه گیری تصادفی طبقه ای برای شرکت در پژوهش انتخاب شدند. داده ها با استفاده از پرسشنامه سواد سلامت دهان، خودکارآمدی بهداشت دهان و مقیاس سلامت دهان و دندان خودگزارش شده جمع آوری و با آزمون همبستگی پیرسون و تحلیل رگرسیون گام به گام تحلیل شدند. یافته ها: هر دو بعد محاسبه (01/0>p و 47 =r) و درک خواندن (01/0>p و 56 =r) در سواد سلامت دهان و هر دو بعد خودکارآمدی مسواک زدن (01/0>p و 60 =r) و نخ دندان کشیدن (01/0>p و 45 =r) با سلامت دهان و دندان خودگزارش شده در دانشجویان همبستگی معنی دار مثبت داشت. نتایج تحلیل رگرسیون گام به گام نشان داد که در گام اول خودکارآمدی نخ دندان کشیدن (59 =R)، درگام دوم درک خواندن (71 =R)، در گام سوم محاسبه (73 =R) و در گام چهارم خودکارآمدی مسواک زدن (74 =R) قادر به پیش بینی معنی دار سلامت دهان و دندان بودند. در مجموع، این متغیرها قادر به پیش بینی 55 درصد از واریانس سلامت دهان و دندان در دانشجویان بودند (01/0>p). نتیجه گیری: سواد سلامت دهان و خودکارآمدی بهداشت دهان نقش مهمی در پیش بینی سلامت دهان و دندان افراد دارد و با توجه اینکه این متغیرها با آموزش در افراد قابل تغییر هستند، می توان جهت افزایش سلامت دهان و دندان افراد مهارت های سواد سلامت دهان یا خودکارآمدی بهداشت دهان را به آن ها آموزش داد.

Title: بررسی ارتباط مصرف میوه و سبزی با افسردگی، اضطراب و استرس در دانشجویان دانشگاه علوم پزشکی شهید صدوقی یزد

http://tbj.ssu.ac.ir/article-1-1295-fa.html

مقدمه : افسردگی در میان قشرهای گوناگون اجتماع وجود دارد و شاخص مهمی در رابطه با بهداشت روانی محسوب می شود. در این میان سلامت روانی دانشجویان گروه های علوم پزشکی با توجه به اهمیت شغلی آنان و این که کارآیی هر انسان درارتباط با سلامت روانی وروحی او است، از اهمیت ویژه ای برخوردار است. مطالعه ی حاضر با هدف بررسی وضعیت افسردگی، اضطراب و استرس در دانشجویان دانشگاه علوم پزشکی یزد و ارتباط آن با مصرف میوه و سبزی انجام شد. روش بررسی: این مطالعه از نوع توصیفی است که در سال 1391 بر روی 200 نفر از دانشجویان دانشگاه علوم پزشکی شهید صدوقی یزد انجام شد. داده ها با روش نمونه گیری خوشه ای دو مرحله ای و با استفاده از پرسشنامه جمع آوری گردید. سوالات پرسشنامه در 2 قسمت شامل الف: اطلاعات زمینه ای و قسمت دوم شامل پرسشنامه استاندارد 21 DASSبود. برای تجزیه و تحلیل داده ها از نرم افزار 16spss استفاده شد. یافته ها: نتایج نشان داد کسانی که مصرف میوه در آن ها کم بود، درجه افسردگی بالاتری داشتند که این رابطه از نظر آماری معنی دار بود. اما بین اضطراب و استرس با مصرف میوه رابطه معنی داری یافت نشد. از نظر مصرف سبزی، کسانی که مصرف سبزی روزانه آن ها کم بود، درجه بالاتری از افسردگی را داشتند و این رابطه از نظر آماری معنی دار بوده است اما بین اضطراب و استرس با مصرف سبزی رابطه معنی داری یافت نشد. نتیجه گیری: با توجه به پایین بودن میزان مصرف میوه و سبزی در دانشجویان و تاثیری که مصرف آن ها بر بهداشت روان بخصوص حیطه افسردگی دارد، آموزش تغذیه و انجام مطالعات بیشتر جهت یافتن علل و عوامل موثر بر عدم دریافت میوه و سبزی توسط دانشجویان ضروری به نظر می رسد.
